# Mortality Outcomes After Spinal Cord Stimulation: A 10-Year Survival Analysis of 330 Patients With Chronic Neuropathic Pain

**DOI:** 10.1227/neu.0000000000003820

**Published:** 2025-10-23

**Authors:** Antti Johannes Luikku, Antti Martikainen, Tiina-Mari Ikäheimo, Mette Nissen, Mikael von und zu Fraunberg, Henna-Kaisa Jyrkkänen, Jukka Huttunen

**Affiliations:** ‡ Neurosurgery of NeuroCenter, Kuopio University Hospital, Kuopio, Finland;; § Substance Abuse Outpatient Clinic, Kuopio University Hospital, Kuopio, Finland;; ‖ Neurosurgery at Neurocenter, Oulu University Hospital, Oulu, Finland;; ¶ Research Unit of Clinical Medicine, University of Oulu, Oulu, Finland

**Keywords:** Chronic pain, Mortality, Spinal cord stimulation, Pain neuromodulation

## Abstract

**BACKGROUND AND OBJECTIVES:**

Spinal cord stimulation (SCS) is an established but intricate therapy for neuropathic pain. Chronic pain is associated with increased mortality; however, the impact of successful SCS treatment on mortality remains unreported. In this retrospective case-controlled study, we present survival and cause-specific mortality data for patients trialed for SCS.

**METHODS:**

The primary outcome was cause-specific mortality in our cohort of 330 consecutive patients, aged 65 years or younger, referred to Kuopio University Hospital for persistent spinal pain syndrome type 2, complex regional pain syndrome, or other neuropathic pain. An SCS device was implanted in 256 patients but later explanted in 78 cases. To compare mortality, 979 matched controls and causes of death were sourced from a national registry.

**RESULTS:**

During a median follow-up of 9.1 years, 28 patients and 39 matched controls died. The 10-year mortality rate was increased in the trial-only group (hazard ratio [HR] = 2.34; CI 1.11-5.09, *P* = .03) and the explanted SCS group (HR = 3.57; CI 1.83-6.98, *P* < .001), whereas no increased mortality was observed in the permanent SCS group compared with matched controls (HR = 1.41; CI 0.68-2.91, *P* = .35). The most frequent causes of death were malignant neoplasms (27%) and external causes (27%).

**CONCLUSION:**

Increased mortality was observed in patients with a negative SCS trial or explanted SCS devices, whereas patients with successful SCS treatment experienced no excess mortality.

**ABBREVIATIONS:**
HR: hazard ratio
ICD-10: International Statistical Classification of Diseases and Related Health Problems 10th Revision
KUH: Kuopio University Hospital
SCS: spinal cord stimulation.

Chronic neuropathic pain is a challenging condition to treat worldwide, with a population prevalence of up to 10%.^1,2^ Chronic pain conditions have been associated with increased mortality and decreased quality of life.^3-6^ A recent study applying propensity score matching found that chronic pain might modestly increase mortality risk.^7^ However, after adjustments for confounding factors such as demographic and health variables, the results suggest that much of the initial strong association between chronic pain and mortality is attributable to these confounders. This highlights the complex interaction between pain, confounding factors, and mortality. Spinal cord stimulation (SCS) is an established treatment of neuropathic pain, although its mechanism of action is still under research.^8^ The effectiveness of SCS is long-lasting and leads to reduced work absenteeism and disability pensions.^9,10^ However, the effectiveness of SCS has recently been questioned by a sham-controlled trial with a specific waveform, warranting further research as recommended by the Cochrane Institution.^11,12^ Assessing the outcomes of SCS is particularly challenging, as chronic pain is evaluated using patient-reported outcomes. Holistic outcome measures for SCS have been proposed, including assessments of daily function, sleep, and health-related quality of life.^13^

Mortality, in its various forms, is one of the fundamental reported outcomes of any medical treatment regardless of the disease or condition involved.^14^ Although the effects of chronic pain on mortality have been emphasized, the impact of neuropathic pain—and specifically chronic pain treated with SCS—on overall mortality is poorly understood. In this study, we present a retrospective, long-term follow-up of patients treated with SCS for severe neuropathic pain at the Kuopio University Hospital (KUH) Neurosurgery Department. We report the 10-year survival and cause-specific mortality of patients with continuous SCS treatment, discontinued SCS treatment, and failed trials, in comparison with a matched control population. We hypothesize that increased mortality may be observed in patients with chronic neuropathic pain who had negative trials or discontinued SCS treatment. By contrast, due to the intervention and continuous treatment of chronic pain, we expect reduced mortality among patients who continue with SCS treatment.

## METHODS

### Setting

The KUH Neurosurgery Department is the sole neurosurgical facility in Eastern Finland, with a distinct regional responsibility that covered an average population of 800 000 during 1993-2019^15^. KUH is a publicly funded institution, and all SCS devices for the neurosurgical department are currently selected through periodically conducted public tenders. Patients evaluated for SCS were referred to the KUH Neurosurgery neuromodulation team by the KUH pain clinic or by pain specialists from regional hospital pain clinics.

### Patient Selection

**FIGURE 1.**
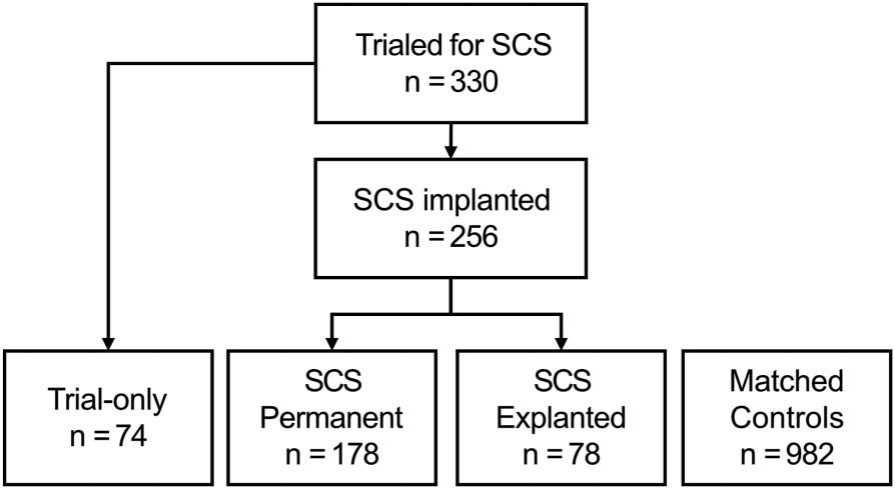
Flowchart for study population. SCS, spinal cord stimulation.

From the regional area of responsibility, a total of 330 consecutive patients were trialed for SCS from 1993 to 2014, forming our retrospective registry (Figure 1). The criteria for an SCS trial included chronic neuropathic pain because of PSPS-T2 (historically referred to as failed back surgery syndrome, complex regional pain syndrome, or other neuropathic pain conditions such as postherpetic neuralgia). For PSPS-T2, the criteria also included radicular pain in 1 or both legs—low back pain alone was not a criterion for trial and was not targeted during SCS implantation or programming. A 1-week trial of an SCS device was conducted, and a permanent SCS device was implanted if patients reported pain relief and perceived improvement in their condition. Electrodes used during the study period are detailed in **Supplementary Table 1** (http://links.lww.com/NEU/F94), with surgical paddle leads predominating. The surgical technique for paddle lead insertion involved either a small medial laminotomy or insertion through the midline via interspinous ligament with a small opening in the ligamentum flavum under general anesthesia. Percutaneous leads were implanted under regional anesthesia, with paresthesia-controlled positioning of the lead. All lead positions were confirmed using fluoroscopy.

### Outcomes of Interest

SCS devices were implanted in 256 patients. During the study period, only tonic stimulation programs were available in the devices used for primary implantation. Follow-up continued until death or the end of 2019. During the follow-up period, patients who underwent SCS device revisions for any reason and were reimplanted early remained categorized within the permanent SCS group. Causes of death were obtained from a Finnish national registry (Finnish Digital and Population Data Services Agency, https://dvv.fi/en). Cohort patients were categorized into 3 groups: the permanent SCS group, the explanted SCS group, and the trial-only group. Three matched controls (based on age [ ±6 months], sex, and birthplace) for each cohort patient were sourced from the Finnish Population Register Center, including causes of death (Finnish Digital and Population Data Services Agency, https://dvv.fi/en). However, 11 study patients had fewer than 3 controls because of unavailability of subjects meeting the set parameters.

### Ethics

All procedures involving human participants adhered to the ethical standards of the institutional and/or national research committees, as well as the 1964 Declaration of Helsinki and its subsequent amendments or comparable ethical standards. This study used retrospective clinical registry data, and individual patient consent was not sought, as the data were collected as part of routine clinical care. The retrospective use of these data was reviewed and approved by the author's institution.

### Statistics

IBM® SPSS® Statistics 29.0 for Windows software was used for statistical analyses. Cohort data are presented as numbers with percentages for categorical variables and as medians with ranges for continuous variables. Comparisons between groups for electrode types and causes of death were performed using the Fisher-Freeman-Halton exact test, including post hoc comparisons. Otherwise, χ^2^ tests were used for categorical variables and Kruskal-Wallis test were used for continuous variables. Kaplan-Meier survival analysis was used to study mortality, and Cox regression was used for group comparisons. *P* values of <.05 were considered statistically significant across all analyses.

## RESULTS

### Primary Outcome

**FIGURE 2.**
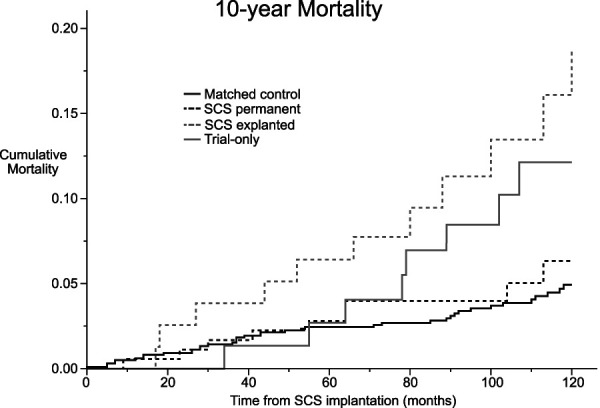
10-year cause-specific cumulative mortality among study groups. SCS, spinal cord stimulation.

A negative 1-week trial with SCS was conducted for 74 patients. During the follow-up, SCS devices were explanted from 78 patients. Over a median follow-up period of 9.1 years, a total of 28 patients (8.5%) in the cohort died, compared with 39 (4.0%) in the control group (Table 1). In Kaplan-Meier survival analysis, the estimated mean survival time was 115.0 months (95% CI: 111.3, 118.6) for the trial-only group, 111.7 months (95% CI: 106.2, 117.3) for the SCS explanted group, 116.6 months (95% CI: 114.2, 119.0) for the SCS permanent group, and 117.2 months (95% CI: 116.3, 118.2) for the control group (Figure 2). In Cox regression analysis, adjusted for age at the time of implantation and sex, the hazard ratio (HR) was 2.34 (95% CI: 1.11, 5.09, *P* = .03) for the trial-only group, 3.57 (95% CI: 1.83, 6.98, *P* < .001) for the SCS explanted group, and 1.41 (95% CI: 0.68, 2.91, *P* = .35) for the SCS permanent group compared with the control group (Table 2). The HR for sex was 0.61 (95% CI: 0.37, 1.01, *P* = .53), favoring women, whereas the HR for age was 1.07 (95% CI: 1.04, 1.10, *P* < .001).

**TABLE 1.** Characteristics of Study Cohort and Matched Control Population

Characteristic	Trial-only	Explanted	Permanent	Control	*P*-value^a,b^
Sample size, n	74	78	178	979	
Sex, n (%)					.11
Male	47 (63.5)	39 (50.0)	83 (46.6)	502 (51.3)	
Female	27 (36.5)	39 (50.0)	95 (53.4)	477 (48.7)	
Survival at the end of follow-up, n (%)					<.001
Alive	66 (89.2)	67 (85.9)	169 (94.9)	940 (96.0)	
Deceased	8 (10.8)	11 (14.1)	9 (5.1)	39 (4.0)	
Surgical characteristics					
Median age (range) at implantation in years	49.0 (19-65)	48.5 (20-65)	48.0 (22-65)	n/a	.96
Diagnosis, n (%)					.10
PSPS-T2	47 (63.5)	41 (52.6)	121 (68.0)	n/a	
CRPS	8 (10.8)	6 (7.7)	10 (5.6)	n/a	
Other neuropathic pain	19 (25.7)	31 (39.7)	47 (26.4)	n/a	

CRPS, complex regional pain syndrome, n/a, not available; PSPS T-2, persistent spinal pain syndrome type 2.

a *P*-value compares the following groups: trial-only, explanted, permanent, and control.

b Chi-square test was used to compare proportions and Kruskal-Wallis test to compare medians.

**TABLE 2.** Results of the Cox Proportional Hazard Model for Survival Among Study Groups

Study Group	Sample size, n	HR^a^	95% CI^a^	*P*-value^b^
Trial-only	74	2.34	1.11-5.09	.03
Explanted	78	3.57	1.83-6.98	<.001
Permanent	178	1.41	0.68-2.91	.35
Control	979	reference	n/a	n/a

HR, hazard-ratio; n/a, not available.

^a^Cox-regression adjusted for age at the time of implantation and sex.

b *P*-value compares trial-only, explanted, permanent, with the control group as the reference group.

### Secondary Outcomes

**FIGURE 3.**
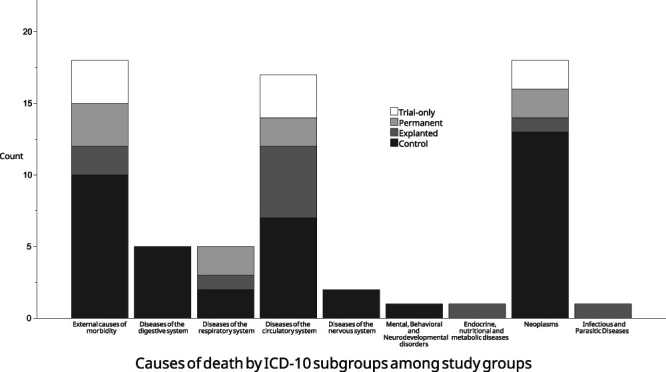
Bar plot for ICD-10 subgroups for causes of death among study groups. ICD-10 subgroups containing at least 1 cohort patient are shown. ICD-10, International Statistical Classification of Diseases and Related Health Problems 10th Revision.

The most frequent International Statistical Classification of Diseases and Related Health Problems 10th Revision (ICD-10) subgroups for causes of death were external causes of morbidity, neoplasms, and diseases of the circulatory system (Figure 3) (Table 3). No statistically significant differences were observed in causes of death among the study groups.

**TABLE 3.** Causes of Death by ICD-10 Subgroups Among Study Groups

Cause of death	Trial-only	Explanted	Permanent	Control	Total	*P*-value^a,b^
Sample size, n	8	11	9	39	67	
Cause of death ICD-10 subgroup^c^, n (%)						.38
Certain infectious and parasitic diseases	0 (0.0)	1 (9.1)	0 (0.0)	0 (0.0)	1 (1.5)	
Neoplasms	2 (25.0)	1 (9.1)	2 (22.2)	13 (32.3)	18 (26.5)	
Endocrine, nutritional, and metabolic diseases	0 (0.0)	1 (9.1)	0 (0.0)	0 (0.0)	1 (1.5)	
Mental and behavioural disorders	0 (0.0)	0 (0.0)	0 (0.0)	1 (2.6)	1 (1.5)	
Diseases of the nervous system	0 (0.0)	0 (0.0)	0 (0.0)	2 (5.1)	2 (2.9)	
Diseases of the circulatory system	3 (37.5)	5 (45.5)	2 (22.2)	7 (17.9)	17 (25.0)	
Diseases of the respiratory system	0 (0.0)	1 (9.1)	2 (22.2)	2 (5.1)	5 (7.4)	
Diseases of the digestive system	0 (0.0)	0 (0.0)	0 (0.0)	5 (12.8)	5 (7.4)	
External causes of morbidity and mortality	3 (37.5)	2 (18.2)	3 (33.3)	9 (23.1)	18 (26.5)	

ICD-10, International Statistical Classification of Diseases and Related Health Problems 10th Revision.

a *P*-value compares the following groups: trial-only, explanted, permanent, and control.

b Fisher-Freeman-Halton exact test was used to compare proportions.

c ICD-10 subgroups containing at least 1 cohort patient are shown.

Reasons for SCS device explantation are provided in Table 4. The most common reasons for explantation were loss of efficacy, unneeded device, and infection.

**TABLE 4.** Reasons for Device Explantation During Study Period

Reason for explantation	Sample size, n (%)
Loss of efficacy	54 (69.2)
No need for device	6 (7.7)
Infection	5 (6.5)
Need for MRI (incompatibility)	3 (3.8)
Wrong area of stimulation	3 (3.8)
IPG out of battery	2 (2.6)
IPG pocket pain	2 (2.6)
Other patient-related reason	2 (2.6)
Lead migration	1 (1.3)

IPG, impulse generator.

## DISCUSSION

We present the first report on the potential effects of successful SCS treatment on mortality, comparing patients with chronic neuropathic pain who underwent negative SCS trials, discontinued SCS treatment, or were in matched control group. Increased mortality was observed in patients with negative SCS trials or explanted SCS devices compared with the control group. However, this excess mortality was absent in patients who continued with SCS treatment, suggesting a normalized survival prognosis through successful SCS therapy. No differences in causes of death were found between groups.

Chronic pain has been linked to increased mortality in previous studies.^3-5,16-19^ Cause-specific studies have identified an elevated risk of death from cancer, neoplasms, diseases of the circulatory and respiratory systems, and suicide in chronic pain patients.^18^ In our study, these same ICD-10 cause-of-death categories were present but without statistical significance, likely due to the low number of cases in each category. Increased mortality seems to be tied to lifestyle risk factors such as poor diet, low physical activity, high body mass index, and smoking^17^ Successful SCS treatment may alleviate chronic pain and reduce psychological distress, thereby enabling potential lifestyle changes and rehabilitation.^17-19^ SCS is generally used as a third- or fourth-line treatment for chronic neuropathic pain, suggesting that patients trialed for SCS represent a population with severe, treatment-resistant chronic pain.^20^ Indeed, no excess mortality was observed for musculoskeletal pain or complaints.^21^ This underlines the relevance and impact of our finding of normalized mortality in this challenging patient population, implying that patients with continuous SCS treatment—in contrast to those with failed trials or explanted devices—truly obtain a clinically meaningful improvement in their chronic pain.

Strong opioids are often an alternative to SCS for managing chronic neuropathic pain.^20^ However, prolonged opioid use has severe adverse effects, including association to benzodiazepine use and, most critically, higher all-cause mortality.^22-26^ Our previous findings showed elevated opioid use among patients with negative SCS trials or explanted devices, likely contributing to higher mortality in these groups.^27^ This highlights a key difference: While opioids increase mortality, SCS normalizes it, in line with control groups. Importantly, no deaths in our study were attributable to surgical complications.

Loss of efficacy was the leading cause of explantation in 69% of cases within the SCS explanted group. This challenge, inherent to SCS treatment, is not fully understood.^28^ Proposed causes include neural plasticity and fibrosis around the lead.^29^ Although initial positive responses were noted during trials, heightened expectations may have influenced subjective perceptions of pain relief. Program rotation—commonly suggested to combat loss of efficacy—was less accessible during the study period because of reliance on conventional tonic programs.^28,30^ Alternatives, such as stimulation holidays, show promise but were not widely available at the time.^31^ Nevertheless, comprehensive program adjustments were implemented before explantations when loss of efficacy was detected.

Overall, patients with continuous SCS treatment showed improved outcomes compared with the trial-only and explanted SCS groups. Our previous work demonstrated reduced benzodiazepine use, work absenteeism, and disability pensions in this population.^10,32^ These findings highlight the broader benefits of successful SCS treatment, extending beyond pain relief to improved functional outcomes. Although pain alleviation remains a primary goal, the future direction for SCS outcome measures is holistic and multidimensional.^13^ The normalization of excess mortality observed in our study underscores the need for comprehensive measurement practices.

### Limitations

The observed increase in mortality in the trial-only and explanted SCS groups seems to be multietiological, unrelated to any single cause of death or ICD-10 subgroup. Despite our robust sample size, the number of cases for specific causes of death was small. Future confirmation of our results will require larger, multicenter, and multinational registries. Similar findings of elevated mortality in chronic pain patients have been reported without accounting for SCS treatment.^18^ Strengths of our study include its long follow-up duration, reliable cause-of-death data from a high-quality national registry, and a large, matched control population. More sophisticated methods of control population matching, such as propensity score analysis, should be used in future studies to validate our findings. In addition, as a retrospective registry-based study, it has inherent limitations. Detailed patient-reported outcome measures were unavailable in our older registry, and actual device usage or uptime was not controlled.

### Future Directions

The relationship between holistic outcomes in SCS treatment and mortality should be explored in a detailed, prospective setting. The underlying mechanisms linking successful SCS treatment to reduced mortality remain unknown and warrant more sophisticated research designs.

## CONCLUSION

Patients with negative SCS trials or explanted SCS devices showed increased mortality compared with the control population. By contrast, we report a phenomenon in which successful SCS treatment is associated with the normalization of excess mortality caused by chronic neuropathic pain.

### Data availability

The datasets used and/or analysed during the current study are available from the corresponding author upon reasonable request and in accordance with Finnish data protection laws.

## Supplementary Material

## SUPPLEMENTAL DIGITAL CONTENT

**Supplementary Table 1**. Electrodes used in spinal cord stimulation during study period.
