# Supplementary material for: Mortality Outcomes After Spinal Cord Stimulation: A 10-Year Survival Analysis of 330 Patients With Chronic Neuropathic Pain
Source: Neurosurgery. 2025 Oct 23;99(1):202–8. doi: 10.1227/neu.0000000000003820 (PMC13236027; doi:10.1227/neu.0000000000003820)
Supplement: Supplementary file 1 [file neu-99-202-s001.docx]

SUPPLEMENTARY

Supplementary Table 1: Electrodes used in spinal cord stimulation during study period

|  | Trial–only | Explanted | Permanent | p-value^a,b^ |
| --- | --- | --- | --- | --- |
| Sample size, n (%) | 74 | 78 | 178 |  |
| Electrode |  |  |  | .02 |
| Medtronic Symmix | 58 (78.4) | 60 (76.9) | 126 (70.8) |  |
| Medtronic Specify 5-6-5 | 3 (4.1)^c^ | 6 (7.7)^d^ | 32 (18.0)^c,d^ |  |
| Medtronic Pisces Quad | 0 (0.0) | 1 (1.3) | 3 (1.7) |  |
| Medtronic Specify 2 x 4 | 3 (4.1) | 4 (5.1) | 6 (3.4) |  |
| Medtronic Vectris | 1 (1.4) | 1 (1.3) | 2 (1.1) |  |
| Medtronic Resume TL/II | 9 (12.2) | 4 (5.1) | 9 (5.1) |  |
| Other | 0 (0.0) | 2 (2.6) | 0 (0.0) |  |

a: p-value compares the following groups: trial–only, explanted, and permanent

b: Fisher-Freeman-Halton exact test was used to compare proportions

c: difference in post–hoc Fisher's exact test at p < .05 level between trial-only and permanent group

d: difference in post–hoc Fisher's exact test at p < .05 level between explanted and permanent group
